# Dual red and near-infrared light-emitting diode irradiation ameliorates LPS-induced otitis media in a rat model

**DOI:** 10.3389/fbioe.2023.1099574

**Published:** 2023-02-22

**Authors:** Yoo-Seung Ko, Eun-Ji Gi, Sungsu Lee, Hyong-Ho Cho

**Affiliations:** ^1^ Department of Otolaryngology-Head and Neck Surgery, Chonnam National University Medical School and Chonnam National University Hospital, Gwangju, Republic of Korea; ^2^ Department of Biomedical Science, College of Medicine, Chonnam National University Graduate School, BK21 PLUS Center for Creative Biomedical Scientists at Chonnam National University, Gwangju, Republic of Korea

**Keywords:** near infrared, otitis media, light emitting diode, inflammation, infection

## Abstract

**Objective:** Otitis media (OM) is an infectious and inflammatory disease of the middle ear (ME) that often recurs and requires long-term antibiotic treatment. Light emitting diode (LED)-based devices have shown therapeutic efficacy in reducing inflammation. This study aimed to investigate the anti-inflammatory effects of red and near-infrared (NIR) LED irradiation on lipopolysaccharide (LPS)-induced OM in rats, human middle ear epithelial cells (HMEECs), and murine macrophage cells (RAW 264.7).

**Methods:** An animal model was established by LPS injection (2.0 mg/mL) into the ME of rats *via* the tympanic membrane. A red/NIR LED system was used to irradiate the rats (655/842 nm, intensity: 102 mW/m^2^, time: 30 min/day for 3 days and cells (653/842 nm, intensity: 49.4 mW/m^2^, time: 3 h) after LPS exposure. Hematoxylin and eosin staining was performed to examine pathomorphological changes in the tympanic cavity of the ME of the rats. Enzyme-linked immunosorbent assay, immunoblotting, and RT-qPCR analyses were used to determine the mRNA and protein expression levels of interleukin-1β (IL-1β), IL-6, and tumor necrosis factor-α (TNF-α). Mitogen-activated protein kinases (MAPKs) signaling was examined to elucidate the molecular mechanism underlying the reduction of LPS-induced pro-inflammatory cytokines following LED irradiation.

**Results:** The ME mucosal thickness and inflammatory cell deposits were increased by LPS injection, which were reduced by LED irradiation. The protein expression levels of IL-1β, IL-6, and TNF-α were significantly reduced in the LED-irradiated OM group. LED irradiation strongly inhibited the production of LPS-stimulated IL-1β, IL-6, and TNF-α in HMEECs and RAW 264.7 cells without cytotoxicity *in vitro*. Furthermore, the phosphorylation of ERK, p38, and JNK was inhibited by LED irradiation.

**Conclusion:** This study demonstrated that red/NIR LED irradiation effectively suppressed inflammation caused by OM. Moreover, red/NIR LED irradiation reduced pro-inflammatory cytokine production in HMEECs and RAW 264.7 cells through the blockade of MAPK signaling.

## Introduction

Otitis media (OM) is the most common cause for a preschool-aged child to visit the hospital and is the most frequent infectious disease that leads to antibiotic prescription worldwide (Rosenfeld et al., 2001; Ahmed et al., 2014). OM causes fever and otalgia and may result in complications such as meningitis or hearing loss (Kwak et al., 2014). Acute otitis media (AOM) is usually related to Eustachian tube dysfunction (Tysome and Sudhoff, 2018). Inflammation in the pharyngeal mucosa can be refluxed into the middle ear through the Eustachian tube. This unwanted secretion in the middle ear may result in an infection and cause AOM. AOM often recurs, resulting in repeated or long-term use of antibiotics.

As OM is often characterized by recurrent intractable infection, several strategies to facilitate treatment have been proposed in addition to antibiotics. Instead of the systemic administration of antibiotics, trans-tympanic local drug delivery by increasing the tympanic membrane permeability has been attempted (Al-Mahallawi et al., 2017; Abdelbary et al., 2019). Biofilm formation is the major issue in intractable OM, and methods for biofilm inhibition have been suggested. The use of anti-DNAB II Fab was reported to reduce eDNA, one of the components of biofilm (Novotny et al., 2021). In addition, the Hydrodebrider system was used to disrupt the biofilm (Abi Hachem et al., 2018). The photosensitizer Chlorin e6 was used to enhance bactericidal activity against biofilm (Luke-Marshall et al., 2020).

Photobiomodulation (PBM) was first discovered by Endre Mester, which is known as “low-level laser therapy” (Mester et al., 1968). Kovacs et al. observed hair growth by applying ruby laser (694 nm) to the skin. Subsequently, they discovered that HeNe laser (632.8 nm) can stimulate wound healing (Kovacs et al., 1974). Currently, similar to lasers, LEDs are used as a light source (de Freitas and Hamblin, 2016). Red (600–700 nm) and near-infrared (NIR) (770–1,200 nm) wavelengths are the most commonly used, which have shown positive biologic effects. The effects of blue and green light have also been investigated; however, there are issues due to the short penetration depth (Hamblin, 2017). PBM has been found to have anti-inflammatory properties with positive effects on wound healing, arthritis, brain and muscle injury healing, inflammatory pain, and autoimmune diseases (Hamblin, 2017). As a non-invasive method, PBM has also shown anti-microbial effects by generating heat (photothermal effect) and reactive oxygen species (ROS) (Han et al., 2021).

In the current study, we investigated the effect of simultaneous red and NIR wavelengths for treating OM. We used a dual red and NIR LED and evaluated its effect on an *in vivo* lipopolysaccharide (LPS)-induced AOM rat model as well as human middle ear epithelial cells (HMEECs) and murine macrophage cells (RAW 264.7) *in vitro*.

## Materials and methods

### Animals and experimental OM model

Sprague-Dawley rats (age, 7–8 weeks old; gender, male; weight, 200–250 g) were purchased from Damul Science (Daejeon, Korea). All rats were provided with adequate food and water. Animal experiments were conducted in strict accordance with the recommendations of the Guide for the Care and Use of Laboratory Animals of Chonnam National University and the protocol approved by the Committee on the Ethics of Animal Experiments of Chonnam National University (CNUHIACUC-20027). All rats were alive during the research period, and normal tympanic membranes were observed prior to LPS injection. In this study, ketamine (100 mg/kg) and xylazine (10 mg/kg) were intraperitoneally injected for the anesthesia. Anesthesia were performed before LPS injection or LED irradiation. Animals were also anesthetized before decapitation for euthanasia. The OM animal model was established by injecting LPS (2.0 mg/ml; L9143, Sigma, St. Louis, MO, United States) from Pseudomonas aeruginosa 10 into the ME of the rats through the tympanic membrane. Intra-tympanic injection was used to deliver LPS since it was the least invasive method compared to other surgical approaches opening the bulla. Rats with PBS injection served as the control. After LPS exposure for 24 h, rats with OM were randomly divided into two groups (Red/NIR LED irradiated group versus none-irradiated group).

### Light source and irradiation

In this study, the continuous-wave dual red and NIR LED irradiation system (HK HEALTHCARE CO., LTD., Korea) with wavelengths of 655 nm and 842 nm consisted of a control module and a battery and was connected to an LED light source with a power cable (Supplementary Figure S1). The LED light source unit was composed of an LED light source and an optical fiber. The power intensity of the LED light was 102 W/m^2^. To examine the therapeutic effect of the red/NIR LED on LPS-induced AOM, rats were irradiated with the red/NIR LED through the ear canal for 30 min for 3 days after LPS injection. Optical fiber (diameter = 3 mm) was tightly placed in the external auditory canal cartilaginous portion. The direction of the optical fiber was monitored to always face the tympanic membrane during the irradiation. Since the optical fiber itself might cause otitis externa, the same optical fiber was place into the external auditory canal (under same anesthesia) without turning on the LED light for the control LPS group. The red/NIR LED system for cell experiments consisted of a power connector and a power supply unit, and the upper LED light source was composed of six LEDs. HMEECs and RAW 264.7 cells were irradiated with red and NIR wavelengths of 653 nm and 842 nm, respectively, and an intensity of 49.4 mW/m^2^ for 3 h after LPS stimulation.

### Cell lines

HMEECs (ScienCell, Carlsbad, CA, United States) and murine macrophage RAW 264.7 cells (Korean Cell Line Bank, Seoul, Korea) were grown with EPiCM-2 and RPMI 1640, respectively. HMEECs and RAW 264.7 cells were treated with LPS at a concentration of 10 μg/mL or 1 μg/mL immediately after serum starvation.

### Histopathological analyses

After the rats were euthanized, tympanic bullae were harvested and fixed in 4% paraformaldehyde for 24 h at 4°C, rinsed with PBS, and decalcified in Calci-Clear Rapid (National Diagnostics, Atlanta, GA, United States) for 5 days. The softened bullae were dehydrated and embedded in paraffin. The paraffin-embedded bullae were sectioned into 7 µm longitudinal sections for staining. The sectioned bullae were deparaffinized, rehydrated, and stained with hematoxylin and eosin (H&E) to visualize the ME mucosa.

### Immunohistochemistry

Immunohistochemical detection of myeloperoxidase (MPO), 7 µm-thick sections were deparaffinized and rehydrated. Citrate buffer solution was used for antigen retrieval, followed by incubation at room temperature for 10 min using 0.3% hydrogen peroxide and the removal of the endogenous peroxidase. The sections were incubated with a primary antibody at 4°C overnight and horseradish peroxidase was then applied for 1 h. Signals were developed in 3,3′-diaminobenzidine (DAB) tetrahydrochloride solution containing 0.1% H_2_O_2_ and observed under the microscope. The number of positive MPO neutrophils in each group was calculated by randomly selecting six fields of view at magnification ×40.

### Real-time polymerase chain reaction (qPCR)

Total RNA was extracted from the ME mucosa of rats or cells using the TRIzol reagent (Invitrogen, Carlsbad, CA, United States) following the manufacturer’s instructions. Extracted RNA was used to synthesize complementary DNA with a reverse transcription kit (Takara, Kyoto, Japan). qPCR was performed with SYBR Green (Takara) and monitored using Thermal Cycler Dice^®^ Real Time System III (Takara). Primers were designed to target endogenous genes, and glyceraldehyde 3-phosphate dehydrogenase (*GAPDH* and *Gapdh*) or β-actin (*ACTB* and *Actb*) was used as the endogenous control. The crossing point of target genes with *GAPDH* and *Gapdh* was calculated using formula 2—(target gene − *GAPDH* and *Gapdh or ACTB* and *Actb*), and the relative amounts were quantified. The qPCR primers are listed in Supplementary Table S1.

### Antibodies and western blotting

IL-1β, IL-6, TNF-α, MPO antibodies were purchased from Santa Cruz Biotechnology (Dallas, TX, United States) or Cell Signaling Technology (Danvers, MA, United States). Total ERK, pERK, JNK, pJNK, p38, pp38 (Cell Signaling Technology), and actin (Sigma) were used with the appropriate secondary antibodies from MBL (Shirley, NY, United States). For western blotting, proteins were separated by 12% PAGE and then electrophoretically transferred onto PVDF membranes. The membranes were incubated with primary antibodies, which were diluted according to the manufacturer’s instructions at 4°C overnight. Horseradish peroxidase-conjugated anti-mouse or anti-rabbit IgGs were added as secondary antibodies. The blots were reprobed with anti-actin antibody as the loading control. Finally, immunoreactive proteins were visualized using an enhanced chemiluminescence (ECL) protocol. The protein levels of IL-1β, IL-6, and TNF-α were measured by densitometry, and the relative protein levels were compared with that of actin and depicted as bar graphs (mean ± SEM, *n* = 9).

### ELISA

The concentrations of the cytokines IL-1β, IL-6, and TNF-α in the tympanic bullae of rats were determined by ELISA using human ELISA kits from R&D Systems (Minneapolis, MN, United States), mouse ELISA kits from Elabscience (Houston, TX, United States), and rat ELISA kits from MyBioSource (San Diego, CA, United States). The results are expressed as picograms per milliliter in accordance with the manufacturer’s instructions. The results for the samples following triplicate experiments are depicted as bar graphs (mean ± SEM, n = 3).

### Cell viability assay

The cell viability of HMEECs and RAW 264.7 cells was analyzed using the EZ-Cytox cell viability assay kit from DoGenBio (Seoul, Korea). In brief, the cells were plated and cultured in 96-well plates (5 × 10^3^ cells/well). Next, 10 µL of EZ-Cytox reagent was added to each well and incubated at 37°C for 2 h, and cell viability was examined. The absorbance was measured using an ELISA microplate reader with a wavelength of 450 nm.

### Statistical analysis

We determined the statistical significance of differences by non-parametric analysis, Mann-Whitney U and Kruskal Wallis tests. When the data met normal distribution (among sample size n = 9), one-way ANOVA with post-hoc Tukey HSD or Student’s t-test was performed. Assessment of normality of data was done using Kolmogorov-Smirnov and Shapiro-Wilk test *P* values less than 0.05 were regarded as statistically significant. Statistical analysis was performed using SPSS 27(Chicago, IL, United States).

## Results

### Establishment of LPS-induced AOM in rats

**FIGURE 1:**
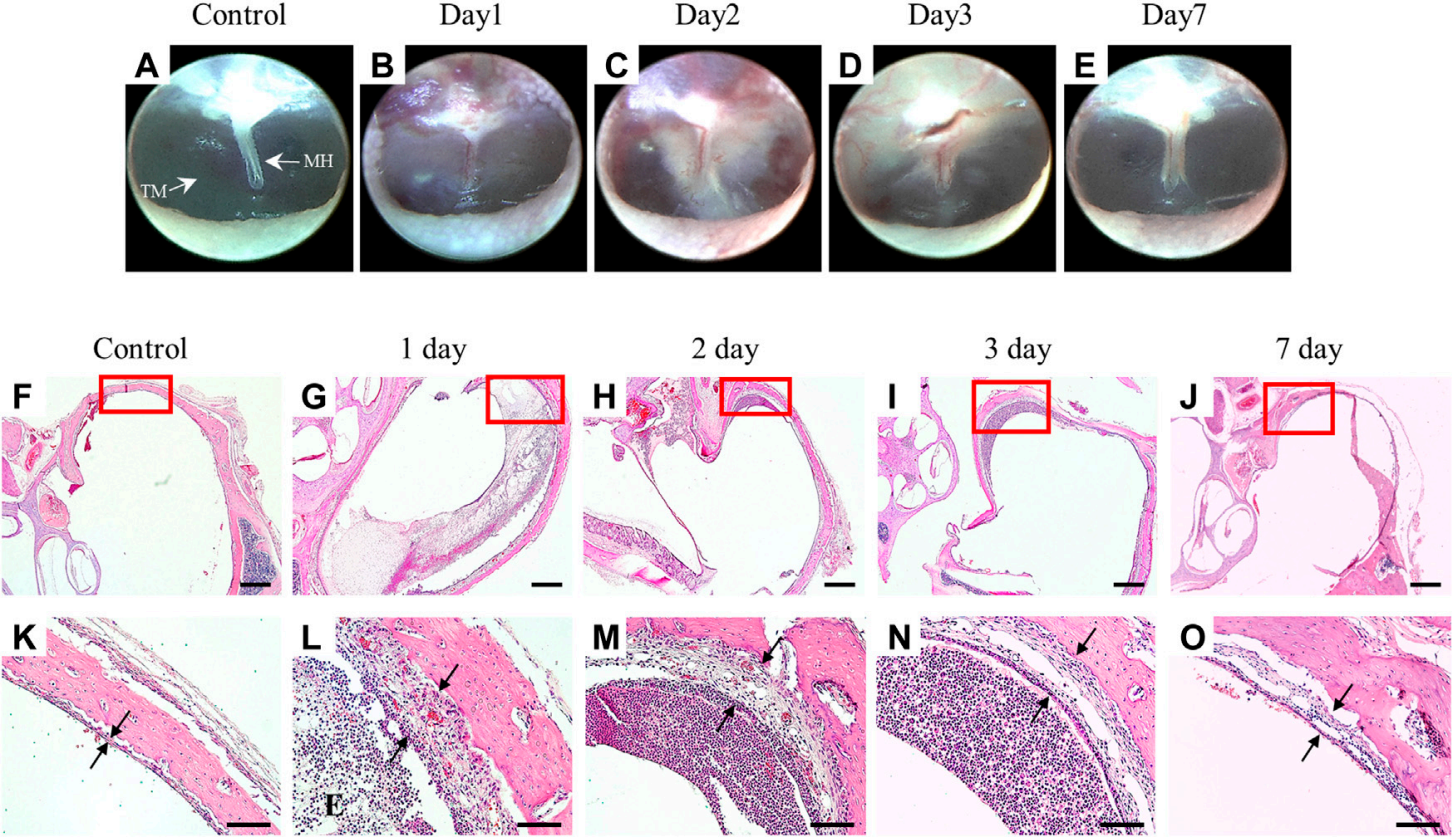
Establishment of a rat model of OM induced by LPS injection through the tympanic membrane. Otoscope images of the tympanic membrane after LPS injection **(A–E)**. ME mucosa stained with H&E, magnification ×2.5 **(F–J)** and ×20 **(K–O)**. Scale bars indicate 500 µm **(F–J)** and 100 µm **(K–O)**. MH: malleus handle, TM: tympanic membrane, E: exudates.

To verify the successful establishment of LPS-induced AOM in a rat model, we observed the tympanic membrane by otoscopy and the histologic sections by H&E staining. As shown in Figures 1A–E, the tympanic membrane of the normal control group was transparent, and there were no signs of an inflammatory reaction; however, the LPS-treated group showed an inflammatory response at 2 days after LPS treatment, and the inflammation disappeared after 7 days. The ME mucosal thickness was significantly increased on the first day after LPS treatment and recovered after 7 days (Figures 1F–O).

### Reduction of ME mucosal thickness by red/NIR LED irradiation

**FIGURE 2:**
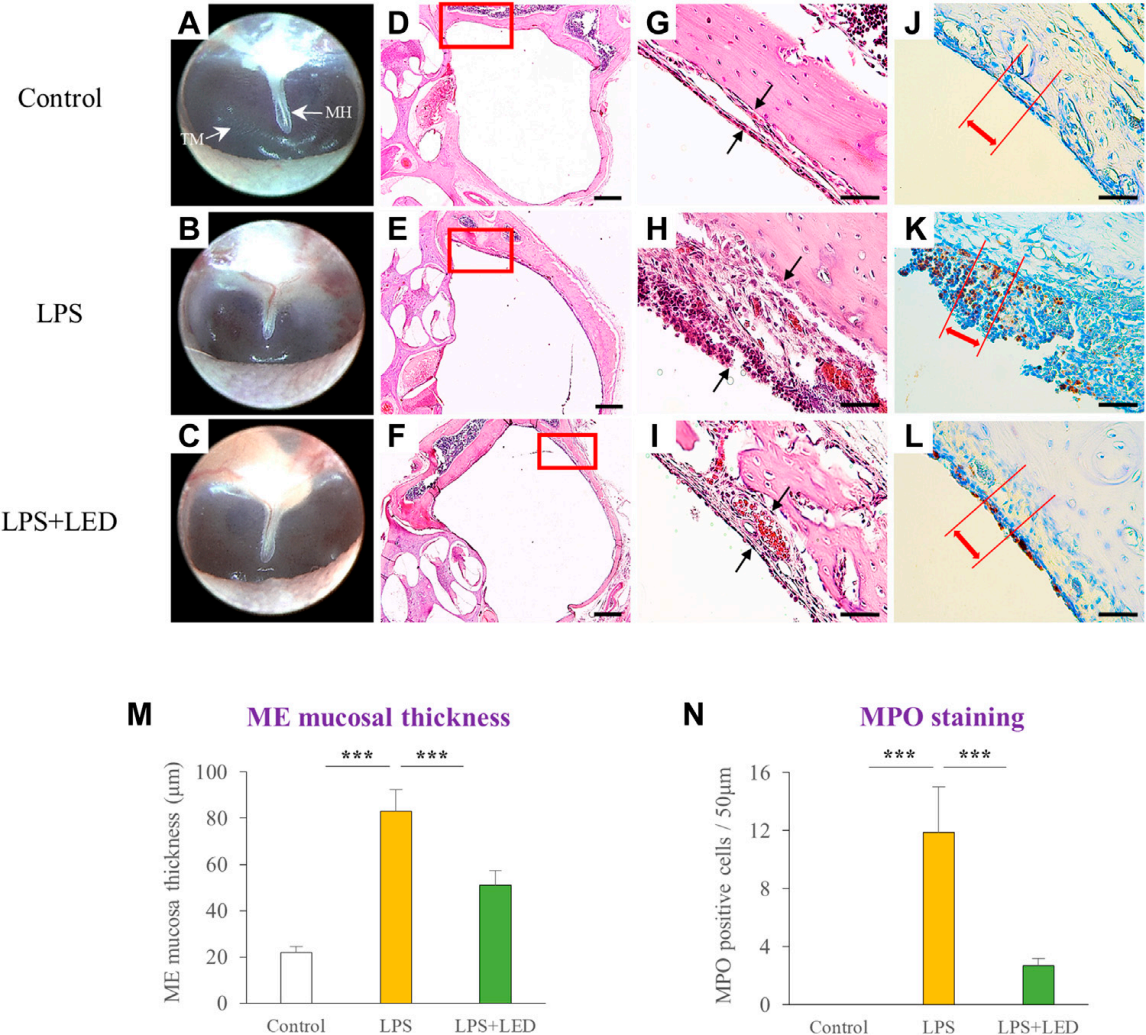
Reduction of ME mucosal thickness by LED irradiation. Representative otoscope images of the tympanic membrane of LPS-treated rats with or without LED irradiation on day 3 **(A–C)**. Representative H&E staining images of the ME histopathology of LPS-treated rats with or without LED irradiation on day 3, magnification ×2.5 **(D–F)** and ×40 **(G–I)**. Scale bars represent 500 µm **(D–F)** and 50 µm **(G–I)**. Immunohistochemical detection of MPO positive cells in LPS-treated rats with or without LED irradiation on day 3 (no positive cells infiltration was observed in the control) **(J–L)**. Scale bars represent 50 µm **(J–L)**. Histograms of the ME mucosal thickness measured in chosen areas (n = 9) **(M)**. One-Way Anova: *p < 0.05, **p < 0.01, and ***p < 0.001. Corresponding quantification of MPO positive cells in chosen areas (n = 9) (N). Kruskal Wallis test: (p) = 0.00006. Mann-Whitney U test: *p < 0.05, **p < 0.01, and ***p < 0.001. Data are expressed as the mean ± SEM. MH: malleus handle, TM: tympanic membrane.

To investigate the therapeutic effect of the red/NIR LED on LPS-induced AOM, rats were irradiated through the ear canal using the red/NIR LED with wavelengths of 655 nm and 842 nm and an intensity of 102 mW/m^2^ for 3 days after LPS injection. As shown in Figures 2A–C, otoscopy revealed that red/NIR LED irradiation reduced the LPS-induced inflammation of the tympanic membrane. In comparison with the control group, the ME mucosal thickness of the LPS-treated group with OM was much thicker with increased inflammatory cell deposits (Figures 2D–I). Compared with the control group, higher degree of MPO positive cell infiltration was observed in the LPS-treated group with OM (Figures 2J–L). The ME mucosal thickness of the control group was 21.5 ± 3.53 μm, which was increased to 102.2 ± 17.81 μm in the LPS-treated OM group. On the other hand, the ME mucosal thickness was significantly reduced to 40.7 ± 8.82 μm in the red/NIR LED-irradiated OM group (Figure 2M). The number of MPO positive neutrophils was significantly decreased in the red/NIR LED-irradiated OM group (Figure 2N). The results indicated that LPS induced AOM in the rats, and the increase in the ME mucosal thickness and inflammatory cell deposits was markedly reversed by red/NIR LED irradiation.

### Reduced expression of pro-inflammatory cytokines in rats with LPS-induced AOM following red/NIR LED irradiation

**FIGURE 3:**
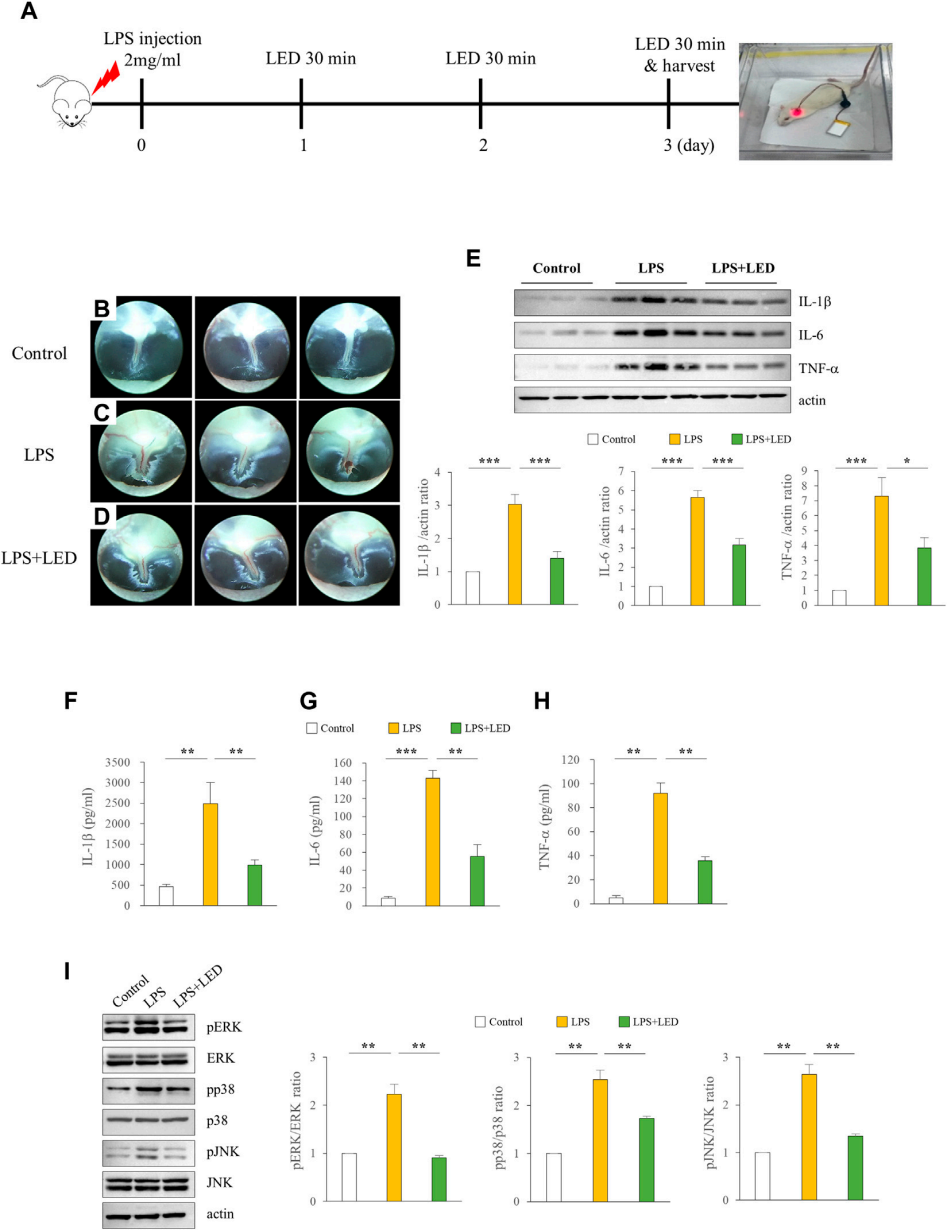
Amelioration of LPS-induced inflammation in the ME of rats by LED irradiation *via* blockade of MAPK signaling. LED irradiation reduced pro-inflammatory cytokine release in LPS-induced OM rats. Experimental protocol for LPS exposure in a rat model of OM (50 μL emulsion of 2 mg/ml LPS) **(A)**. Representative images of the tympanic membrane of LPS-treated rats with or without LED irradiation **(B–D)**. Immunoblot analysis of the expression levels of IL-1β, IL-6, and TNF-α in the tympanic bullae of rats. IL-1β, IL-6, and TNF-α ·protein levels were quantified following immunoblotting (n = 9) **(E)**. One-Way Anova: *p < 0.05, **p < 0.01, and ***p < 0.001. ELISA of pro-inflammatory cytokines including IL-1β **(F)**, IL-6 **(G)**, and TNF-α **(H)** in the tympanic bullae of rats (n = 3). Kruskal Wallis test: (*p*) = 0.001. Reduction of pro-inflammatory cytokines in the tympanic bullae of rats by LED irradiation through the blockade of MAPK signaling (n = 6) **(I)**. Kruskal Wallis test: (*p*) = 0.001. Mann-Whitney U test: *p < 0.05, **p < 0.01, and ***p < 0.001. Data are expressed as the mean ± SEM.

To investigate the in vivo effects of red/NIR LED irradiation on LPS-induced AOM in rats, we designed an experiment as shown in Figure 3A. The inflammatory response is known to be crucial in ME damage under various conditions, such as bacterial exposure (Ferenbach et al., 2007). Therefore, we attempted to examine whether OM amelioration by the red/NIR LED is associated with inflammation suppression and analyzed the expression of pro-inflammatory cytokines by western blotting and ELISA in the tympanic bullae obtained from rats after LPS treatment. The results showed that the increase in inflammation of the tympanic membrane was markedly reduced by LED irradiation (Figures 3B–D), and the protein expression of the pro-inflammatory cytokines IL-1β, IL-6, and TNF-α, which was increased by LPS, was significantly decreased by LED irradiation (Figure 3E). ELISA results were consistent with those of western blotting (Figures 3F–H). OM is associated with ROS levels, and ROS is associated with mitogen-activated protein kinase (MAPK) signaling. Therefore, we subsequently investigated whether MAPK signaling is inhibited by LED irradiation. The phosphorylation of ERK, p38, and JNK, which was increased in the ME tissue of rats after LPS treatment, was inhibited by LED irradiation (Figure 3I). The results demonstrated the efficacy of red/NIR LED irradiation in inhibiting pro-inflammatory cytokine expression induced by LPS, and the inhibition was highly correlated with the suppression of the MAPK signaling pathway.

### Reduced expression of inflammatory cytokines in HMEECs and RAW 264.7 cells following red/NIR LED irradiation

**FIGURE 4:**
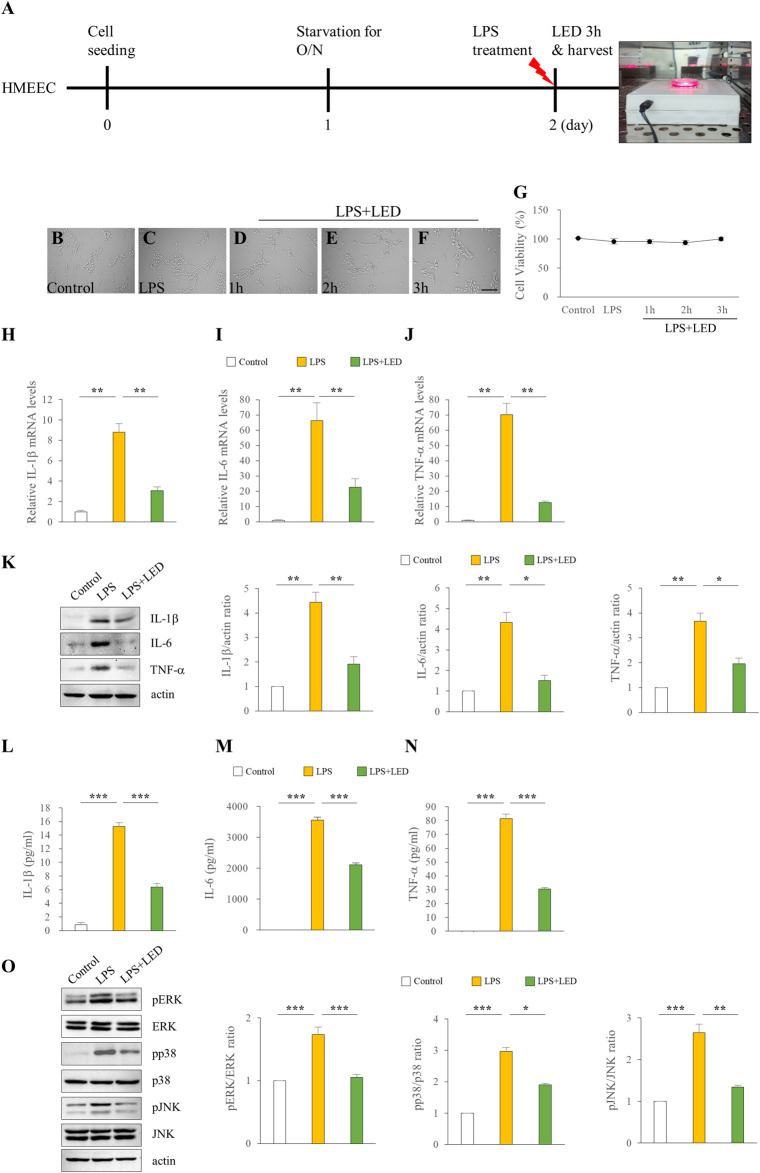
Reduction of the expression of pro-inflammatory cytokines in HMEECs by LED irradiation. Treatment of HMEECs with LPS (50 μg/ml or 10 μg/ml) for 3 h **(A)**. Cell morphology **(B–F)** and viability **(G)** of HMEECs following LED irradiation. The scale bar represents 200 µm **(B–F)**. qPCR assay of the mRNA levels of IL-1β **(H)**, IL-6 **(I)**, and TNF-α **(J)** in HMEECs. Data are presented as the relative fold (n = 6). Kruskal Wallis test: (*p*) = 0.001. Western blotting of the protein expression levels of IL-1β, IL-6, and TNF-β (n = 9) **(K)**. One-Way Anova: *p < 0.05, **p < 0.01, and ***p < 0.001. ELISA of pro-inflammatory cytokines including IL-1β **(L)**, IL-6 **(M)**, and TNF-α **(N)** in HMEECs (n = 3). Kruskal Wallis test: (*p*) = 0.001. Reduction of pro-inflammatory cytokines by LED irradiation through the blockade of MAPK signaling (n = 6) **(O)**. Kruskal Wallis test: (*p*) = 0.002 for pERK and (*p*) = 0.001 for pp38 and pJNK. Mann-Whitney U test: *p < 0.05, **p < 0.01, and ***p < 0.001. Data are expressed as the mean ± SEM.

**FIGURE 5:**
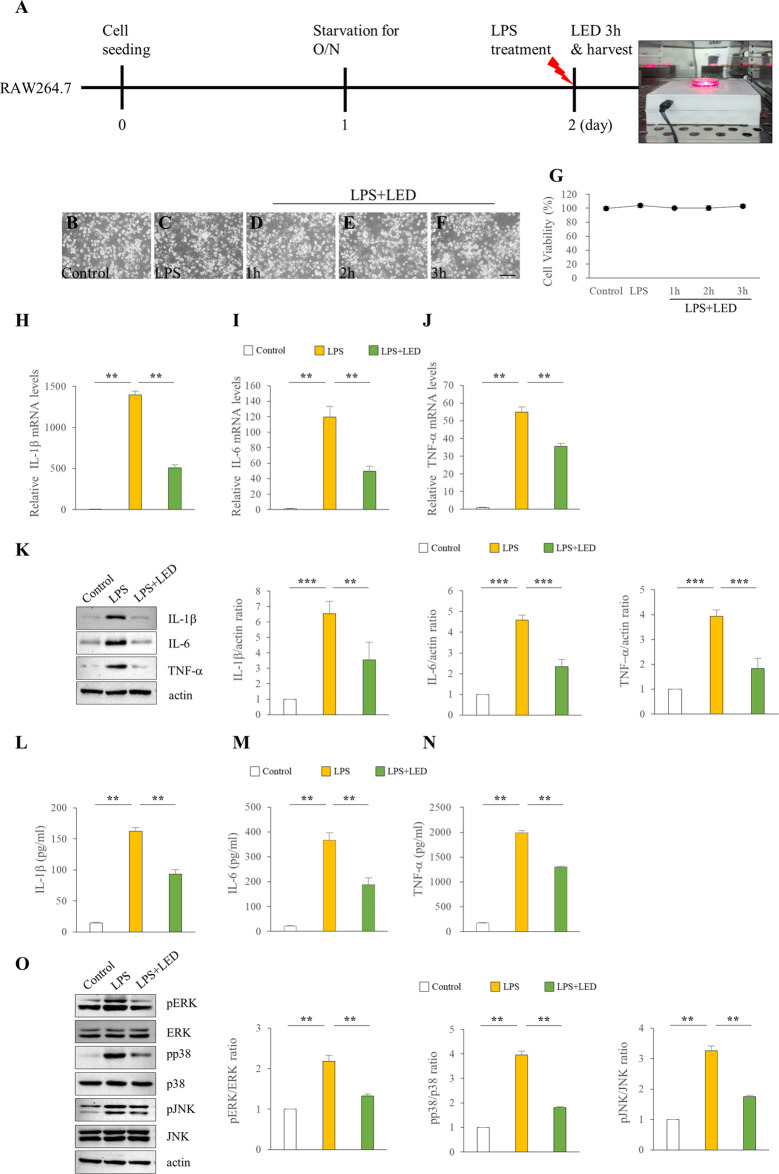
Reduction of the expression of pro-inflammatory cytokines in RAW 264.7 cells by LED irradiation. Treatment of RAW 264.7 cells with LPS (50 μg/ml or 10 μg/ml) for 3 h **(A)**. Cell morphology **(B–F)** and viability **(G)** of RAW 264.7 cells following LED irradiation. The scale bar represents 200 µm **(B–F)**. qPCR assay of the mRNA levels of IL-1β **(H)**, IL-6 **(I)**, and TNF-α **(J)** in RAW 264.7 cells (n = 6). Kruskal Wallis test: (*p*) = 0.001. Data are presented as the relative fold. Western blotting of the protein expression levels of IL-1β, IL-6, and TNF-α (n = 9) **(K)**. One-Way Anova: *p < 0.05, **p < 0.01, and ***p < 0.001. ELISA of pro-inflammatory cytokines including IL-1β **(L)**, IL-6 **(M)**, and TNF-α **(N)** in RAW 264.7 cells (n = 3). Kruskal Wallis test: (*p*) = 0.001. Reduction of pro-inflammatory cytokines by LED irradiation through the blockade of MAPK signaling (n = 6) **(O)**. Kruskal Wallis test: (*p*) = 0.00039 for pERK and pp38 and (*p*) = 0.001 for pJNK. Mann-Whitney U test: *p < 0.05, **p < 0.01, and ***p < 0.001. Data are expressed as the mean ± SEM.

**FIGURE 6:**
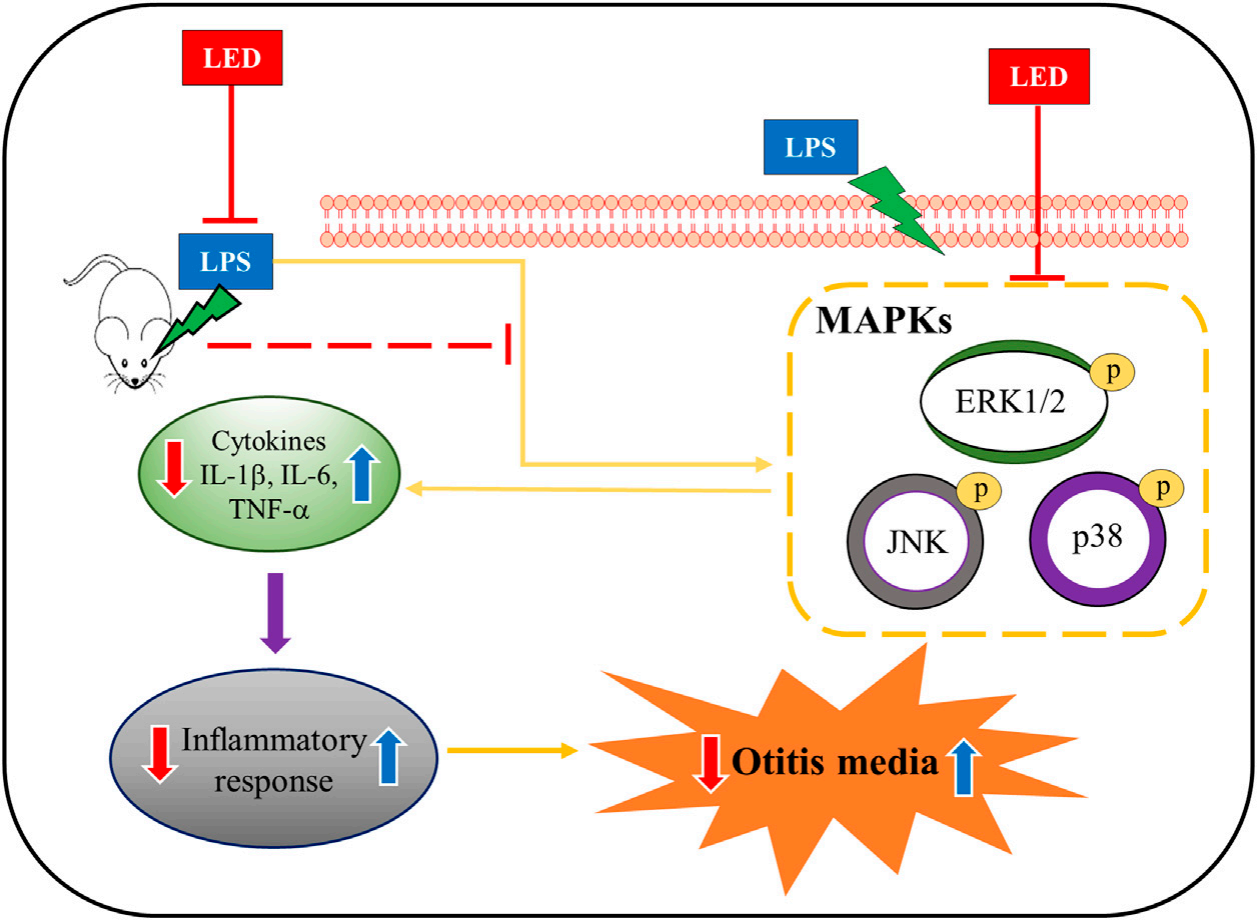
Representative schematic of OM amelioration by LED irradiation. LED reduced the expression of pro-inflammatory cytokines induced by LPS exposure in a rat model of OM, HMEECs, and RAW 264.7 cells *via* the inhibition of MAPK signaling.

We examined the in vitro effect of red/NIR LED irradiation on the expression of pro-inflammatory cytokines. The HMEEC line has been widely used in OM studies; thus, we used this cell line to investigate the effect of LED irradiation on LPS-induced cytokine elevation. The experimental design for LED irradiation with HMEECs is illustrated in Figure 4A. We first confirmed the cell viability of HMEECs after LED irradiation. The number of viable cells did not show any difference over time in the LED-irradiated group compared with the control group (Figures 4B–G). As shown in Figures 4H–J, IL-1β, IL-6, and TNF-α mRNA levels were significantly increased with LPS stimulation at 50 μg/ml for 3 h, which were greatly reduced by LED irradiation in HMEECs. Furthermore, western blot results revealed that the expression levels of IL-1β, IL-6, and TNF-α were increased by LPS exposure, and this upregulation was reversed by LED irradiation in HMEECs (Figure 4K). Additionally, the concentrations of IL-1β, IL-6, and TNF-α were determined by ELISA, which were consistent with the western blot results (Figures 4L–N). The phosphorylation of ERK, p38, and JNK, which was increased after LPS stimulation, was inhibited by LED irradiation in HMEECs (Figure 4O). Important inflammatory mediators in OM are produced by infiltrating immune cells such as macrophages (Juhn et al., 2008). LPS can induce various inflammatory mediators and stimulate local macrophages to produce soluble mediators including IL-1β, IL-6, and TNF-α. Therefore, we investigated the effect of red/NIR LED irradiation on the expression of pro-inflammatory cytokines in RAW 264.7 cells, a mouse macrophage cell line. The experimental design for RAW 264.7 cells was similar to that for HMEECs, as shown in Figure 5A. LED irradiation showed no cytotoxicity towards RAW 264.7 cells (Figures 5B–G). Likewise, the mRNA and protein expression levels of IL-1β, IL-6, and TNF-α, which were increased by LPS treatment, were markedly inhibited by LED irradiation in RAW 264.7 cells (Figures 5H–N). Moreover, the phosphorylation of ERK, JNK, and p38, which was increased by LPS, was attenuated by LED irradiation in RAW 264.7 cells (Figure 5O). These results indicated that red/NIR LED irradiation suppressed the expression of pro-inflammatory cytokines induced by LPS in the macrophage cell line RAW 264.7 through the blockade of MAPK signaling. Overall, our study demonstrated that red/NIR LED irradiation effectively suppressed inflammation caused by OM. Moreover, red/NIR LED irradiation reduced pro-inflammatory cytokine production in HMEECs and RAW 264.7 cells by blocking MAPK signaling (Figure 6).

## Discussion

OM with effusion is a common infectious disease caused by several factors such as viruses, bacteria, and Eustachian tube dysfunction. In OM, the inflammatory process in the ME caused by bacteria involves macrophages and several cytokines (Barzilai et al., 1999). Endotoxins are known to trigger inflammation in the ME and stimulate macrophages to secrete TNF-α and IL-1 (Willett et al., 1998). A previous study has reported that the expression of IL-6 and TNF-α plays an important role in the development of OM in an animal model with effusion (Johnson et al., 1994). In particular, the treatment for OM consists of vaccination and antibiotics. The incorporation of pneumococcal conjugate vaccines has reduced the incidence of OM. However, some serotypes of pneumococci are not covered by the current vaccines, are increasing (Kaur et al., 2017). Moreover, multidrug-resistant pneumococci may lead to recurrent AOM (Gavrilovici et al., 2022). Consequently, finding an effective and safe therapeutic strategy for OM is an ongoing process.

Recently, the efficiency of LED irradiation in increasing tissue blood perfusion, ameliorating post-inflammatory hyperpigmentation, reducing the wound diameter, and promoting the healing of inflammatory conditions such as arthritis has been reported (Dall Agnol et al., 2009; Li et al., 2010; Wu and Persinger, 2011; Kuboyama et al., 2014). Furthermore, LED irradiation was found to decrease the formation of inflammatory cells and the expression of inflammatory cytokines in a collagen-induced Achilles tendonitis rat model (Xavier et al., 2010). The LED device used in this study has a structure that directly irradiates light to the inflamed site of the ME at wavelengths highly effective for inflammation. Phototherapy has been introduced for treating otologic diseases. A 632 nm diode laser effectively killed *S. pneumonia*, *H. influenzae*, and *M. catarrhalis* in OM (Jung et al., 2009). An 808 nm diode laser was used to prevent noise-induced hearing loss (Lee et al., 2019). There are several advantages in using LEDs compared with lasers, such as safety, portability, ability to irradiate large tissue areas at the same time, incorporation of wearable technology, and a much lower cost per mW.

PBM utilizes red and red/IR irradiation to stimulate healing, relieve pain, and reduce inflammation. Previously, LED irradiation has shown anti-inflammatory effects on collagen-induced arthritis in mice (Kuboyama et al., 2014) and *P. gingivalis* LPS-treated human gingival fibroblasts (Choi et al., 2012). In the present study, rats were administrated with LPS to induce OM, and the ability of red/NIR LED irradiation in reducing inflammation was examined. We confirmed that AOM was successfully established in the rats based on the presence of inflammation in the tympanic membrane and the thickened ME mucosa. Red/NIR LED irradiation significantly reduced the inflammatory response in the tympanic membrane and the ME mucosal thickness, demonstrating that red/NIR LED irradiation ameliorated LPS-induced AOM in the ME of the rats.

Pro-inflammatory cytokines regulate the pathogenesis of OM by initiating an inflammatory response to infection and may cause the transformation from acute to chronic OM. Elevated concentrations of IL-1β, IL-6, and TNF-α in children may be correlated with OM; in particular, IL-6, a marker of a bacterial infection, can induce C-reactive protein (CRP) production and appears to play a vital role in inflammation in OM (Yellon et al., 1995). LED irradiation has been found to be effective for the suppression of pro-inflammatory cytokines, suggesting that LED therapy could be an effective strategy in ameliorating diseases associated with inflammation (Nomura et al., 2001; Kuboyama et al., 2014). Therefore, restricting the secretion of pro-inflammatory cytokines may be an effective approach for the treatment of OM. In the present study, the expression levels of IL-1β, IL-6, and TNF-α were significantly increased in the LPS group, which was in agreement with histological findings; red/NIR LED irradiation significantly inhibited the secretion of these pro-inflammatory cytokines, demonstrating that red/NIR LED irradiation could treat OM by impeding pro-inflammatory cytokine release.

Severe diseases can emerge due to an inadequate cytokine response, and cytokines are recognized as regulators in the immunopathology of an ever-increasing number of diseases including OM. Adequate cytokine production is essential for the development of protective immunity (Choi et al., 2012). Therefore, regulation of cytokines is essential for all infectious diseases including OM.

Excessive ROS production is associated with inflammation, aging, and human diseases (Snezhkina et al., 2019). In several human diseases, OM is correlated with ROS levels, and the IL-17 pathway is known to be associated with ROS levels in OM with effusion (Abdelhafeez and Mohamed, 2021). Specifically, ROS is associated with MAPK signaling, which is known as a master regulator of inflammation. JNK and p38 are activated by ROS, and ERK1/2 promotes ROS generation (Genestra, 2007). In agreement with previous findings, we found that the MAPK signaling pathway was activated after LPS stimulation, resulting in the high expression levels of phosphorylated p38, ERK1/2, and JNK, which were reduced by red/NIR LED irradiation. Therefore, the study demonstrated that the amelioration of AOM by LED irradiation may be associated with MAPK suppression.

The use of MAPK inhibitors has been regarded as an attractive treatment strategy because of their ability in reducing both the synthesis and signaling of pro-inflammatory cytokines. In particular, potent anti-inflammatory drugs inhibiting both p38 MAPK and JNK can suppress macrophage inflammatory cytokines, including IL-1β, IL-6, TNF-α, and macrophage inflammatory protein 1α and 1β (Bianchi et al., 1996; Cohen et al., 1996). Taken together, our results showed that red/NIR LED irradiation significantly inhibited pro-inflammatory cytokine levels and MAPK activity upregulated by LPS, suggesting that red/NIR LED irradiation may be an effective method for the treatment of OM.

## Conclusion

In the present study, owing to the anti-inflammatory effects of dual red and NIR LED irradiation, inflammatory cytokines were inhibited in an LPS-induced AOM rat model, as well as HMEECs and RAW 264.7 cells. Moreover, the decrease in pro-inflammatory cytokine production following LED irradiation was mediated by the blockade of MAPK signaling. Collectively, these findings suggest that red/NIR LED irradiation may be an effective therapeutic strategy for the treatment of OM.

## Data Availability

The raw data supporting the conclusions of this article will be made available by the authors, without undue reservation.
