# Corrigendum: Dual red and near-infrared light-emitting diode irradiation ameliorates LPS-induced otitis media in a rat model

**DOI:** 10.3389/fbioe.2025.1591082

**Published:** 2025-05-19

**Authors:** Yoo-Seung Ko, Eun-Ji Gi, Sungsu Lee, Hyong-Ho Cho

**Affiliations:** ^1^ Department of Otolaryngology-Head and Neck Surgery, Chonnam National University Medical School and Chonnam National University Hospital, Gwangju, Republic of Korea; ^2^ Department of Biomedical Science, College of Medicine, Chonnam National University Graduate School, BK21 PLUS Center for Creative Biomedical Scientists at Chonnam National University, Gwangju, Republic of Korea

**Keywords:** near infrared, otitis media, light emitting diode, inflammation, infection

In the published article, there was an error. The light intensity value was incorrect.

A correction has been made to **Abstract**, paragraph 2. This sentence previously stated:

“A red/NIR LED system was used to irradiate the rats (655/842 nm, intensity: 102 mW/m^2^, time: 30 min/day for 3 days and cells (653/842 nm, intensity: 49.4 mW/m^2^, time: 3 h) after LPS exposure.”

The corrected sentence appears below:

“A red/NIR LED system was used to irradiate the rats (655/842 nm, intensity: 163.2 W/m^2^, time: 30 min/day for 3 days and cells (653/842 nm, intensity: 19.76 W/m^2^, time: 3 h) after LPS exposure.”

A correction has been made to **Materials and Methods, Light source and irradiation**, Paragraph 1. This sentence previously stated:

“The power intensity of the LED light was 102 W/m^2^.”

“HMEECs and RAW 264.7 cells were irradiated with red and NIR wavelengths of 653 nm and 842 nm, respectively, and an intensity of 49.4 mW/m^2^ for 3 h after LPS stimulation.”

The corrected sentence appears below:

“The power intensity of the LED light was 163.2 W/m^2^.”

“HMEECs and RAW 264.7 cells were irradiated with red and NIR wavelengths of 653 nm and 842 nm, respectively, and an intensity of 19.76 W/m^2^ for 3 h after LPS stimulation.”

A correction has been made to **Results, Reduction of ME mucosal thickness by red/NIR LED irradiation**. This sentence previously stated:

“To investigate the therapeutic effect of the red/NIR LED on LPS-induced AOM, rats were irradiated through the ear canal using the red/NIR LED with wavelengths of 655 nm and 842 nm and an intensity of 102 mW/m^2^ for 3 days after LPS injection.”

The corrected sentence appears below:

“To investigate the therapeutic effect of the red/NIR LED on LPS-induced AOM, rats were irradiated through the ear canal using the red/NIR LED with wavelengths of 655 nm and 842 nm and an intensity of 163.2 W/m^2^ for 3 days after LPS injection.”

In the published article, the caption for **Supplementary Figure S1** was missing. The correct caption appears below:

“Schematic diagram of the LED system: (a) LED irradiation system for animal experiments. The main body consisted of a control module and a battery connected with a power cable to an LED light source for easy fixing to the animals’ ear. The LED light source unit was composed of an LED light source and an optical fiber. (b) Characteristics of the LED light source for animal experiments. The wavelength of the red LED was 655 nm, and the wavelength of the NIR LED was 842 nm. The power intensity of the LED was 163.2 W/m^2^. (c) LED irradiation system for cell experiments. The main body consisted of a power connector and a power supply unit that allows the insertion of a 5V adapter. The upper LED light source consisted of six LEDs, and the upper part was made flat so that the cell test plate can be irradiated from the lower or upper position. (d) Characteristics of the LED light source for cell experiments. The wavelength of the red LED was 653 nm, and the wavelength of the NIR LED was 842 nm. The power intensity of the LED was 19.76 W/m^2^. Measurements were performed at 0.2 m using Neolite G500 (PIMACS).”

The authors apologize for these errors and state that this does not change the scientific conclusions of the article in any way. The original article has been updated.

