# Supplementary figures and images for: Dual red and near-infrared light-emitting diode irradiation ameliorates LPS-induced otitis media in a rat model
Source: Front Bioeng Biotechnol. 2023 Feb 22;11:1099574. doi: 10.3389/fbioe.2023.1099574 (PMC9992796; doi:10.3389/fbioe.2023.1099574)

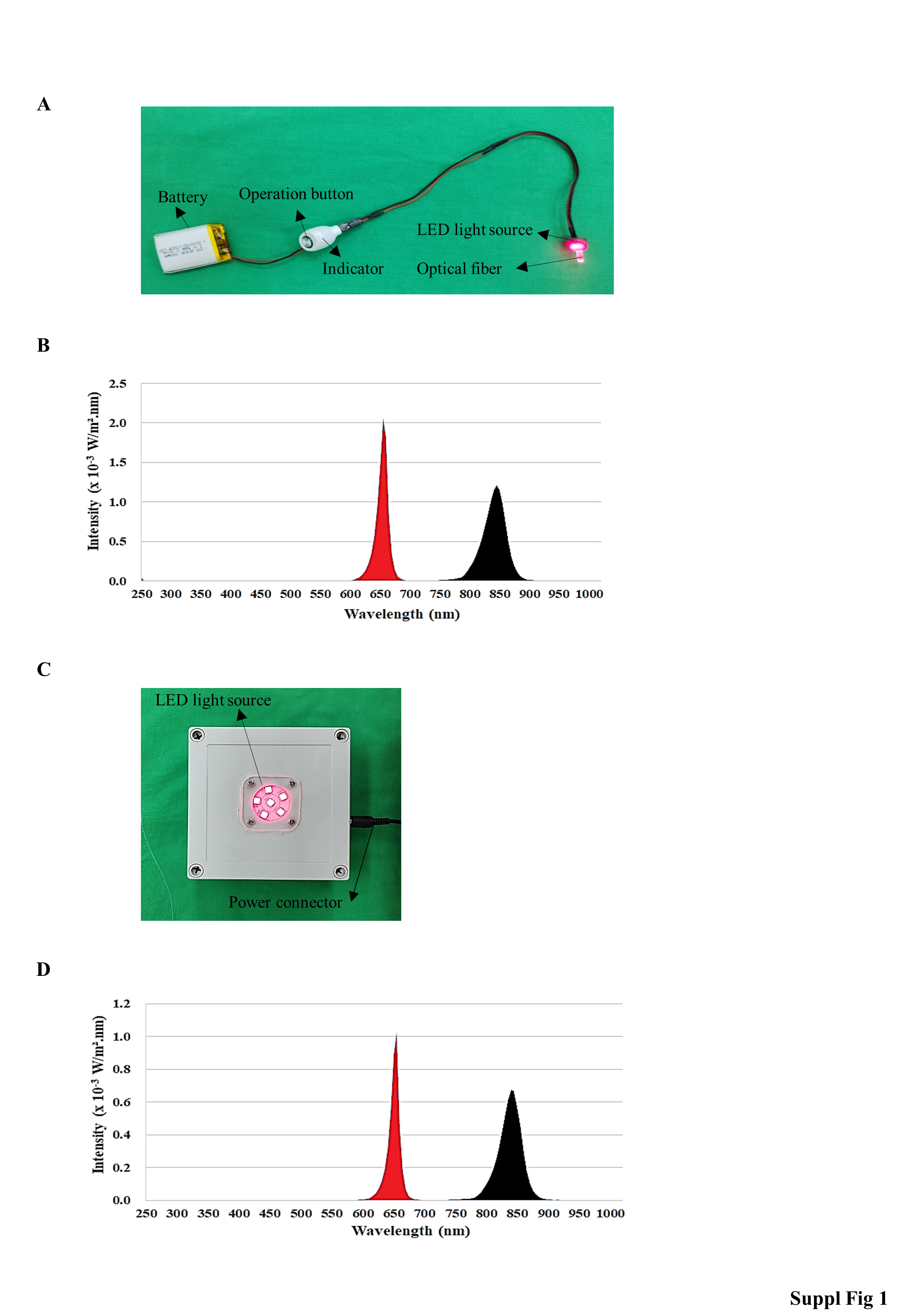

Supplement: Supplementary file 1 [file Image1.tif]
